# Cross-species conservation of complementary amino acid-ribonucleobase interactions and their potential for ribosome-free encoding

**DOI:** 10.1038/srep18054

**Published:** 2015-12-10

**Authors:** John G. D. Cannon, Rachel M. Sherman, Victoria M. Y. Wang, Grace A. Newman

**Affiliations:** 1 Department of Biology, Carleton College, 1 College Street, Northfield MN, 55057, United States; 2 Department of Biology, Harvey Mudd College, 301 Platt Blvd, Claremont CA 91711, United States; 3 Department of Computer Science, Harvey Mudd College, 301 Platt Blvd, Claremont CA 91711, United States; 4 Department of Biochemistry, University of Cambridge, 80 Tennis Court Road, Cambridge, CB2 1GA, United Kingdom; 5 Department of Mathematics, Carleton College, 1 College Street, Northfield MN, 55057, United States

## Abstract

The role of amino acid-RNA nucleobase interactions in the evolution of RNA translation and protein-mRNA autoregulation remains an open area of research. We describe the inference of pairwise amino acid-RNA nucleobase interaction preferences using structural data from known RNA-protein complexes. We observed significant matching between an amino acid’s nucleobase affinity and corresponding codon content in both the standard genetic code and mitochondrial variants. Furthermore, we showed that knowledge of nucleobase preferences allows statistically significant prediction of protein primary sequence from mRNA using purely physiochemical information. Interestingly, ribosomal primary sequences were more accurately predicted than non-ribosomal sequences, suggesting a potential role for direct amino acid-nucleobase interactions in the genesis of amino acid-based ribosomal components. Finally, we observed matching between amino acid-nucleobase affinities and corresponding mRNA sequences in 35 evolutionarily diverse proteomes. We believe these results have important implications for the study of the evolutionary origins of the genetic code and protein-mRNA cross-regulation.

Despite having been discovered more than 50 years ago^1^,^2^ and studied for nearly as long^3^, the evolutionary basis of the universal genetic code remains an elusive challenge^4^. Even before the discovery of messenger RNA (mRNA) and the elucidation of the genetic code George Gamow proposed a stereochemical hypothesis based on the then recently determined structure of DNA^5^. According to Gamow, amino acids could fit into the “holes” and grooves of the double-stranded DNA helix and this would depend on specific affinities between amino acid side chains and the four DNA nucleobases^6^. Later, Carl Woese used chromatographic techniques to show that the codon assignments in the genetic code were non-random (i.e. chemically similar amino acids were coded for by similar codons) and that amino acids had varying propensities for binding nucleobase analogs^7^. Woese further proposed that in a pre-cellular environment “translation” may even have occurred bidirectionally due to so-called “direct templating” between polynucleotides and polypeptides^8^. The stereochemical basis for the genetic code was later investigated further, notably by Michael Yarus^9^, using X-ray crystallography and NMR data of RNA-protein complexes. His investigations revealed that a so-called “polar profile”, the interactions of triplet codons with the polar moieties of the amino acids they encode, explained some *in vivo* RNA-protein binding sites. Furthermore, the Yarus group extended the analysis to include pairs of adjacent amino acids in a polypeptide, arguing that simultaneous recognition of two amino acids may facilitate spontaneous peptide bond formation^10^. To further study the evolution of the modern translation apparatus, Johnson & Wang^11^ investigated ribosomal RNA-amino acid interactions in prokaryotes and found that there were a significant number of interactions between amino acids and their respective anti-codons, possibly providing the gap between direct templating and the RNA adaptor hypothesis^12^.

Other recent studies have shown the potential for physiochemical interactions between polypeptide stretches and their cognate mRNA molecules^13^, and direct auto-regulation between proteins and cognate mRNA has been shown to facilitate alternative splicing^14^ and RNA folding^15^. The formation of large databases of protein-RNA complexes has allowed the inference of amino acid- ribonucleobase interaction affinities from structural information. Previous work has independently shown that these interaction affinities mirror the purine^16^ and pyrimidine^17^ content of corresponding mRNA sequences. However, despite this recent work there remain significant questions concerning the functional importance and conservation of these correlations.

Here we set out to confirm previous findings^13^, upon which the following experiments are primarily based, and to expand our knowledge of amino acid-nucleobase interactions. We utilize the Protein-RNA Interface Database (PRIDB)^18^ to infer amino acid-ribonucleobase affinities and show that these affinities correspond to the purine and pyrimidine content of their respective codons in both the standard and mitochondrial variants of the genetic code. Moreover, we find that calculated interaction affinities can be used to roughly predict protein primary sequence from mRNA. Finally, we show this complementarity is conserved at the level of windowed mRNA sequences and corresponding amino acid sequences across the proteomes of 35 diverse species. These results support an evolutionary role for physiochemical amino acid-ribonucleobase interaction affinities in the establishment of the universal genetic code.

## Results

### Calculation of Amino Acid-Nucleobase Interaction Preferences

**Figure 1:**
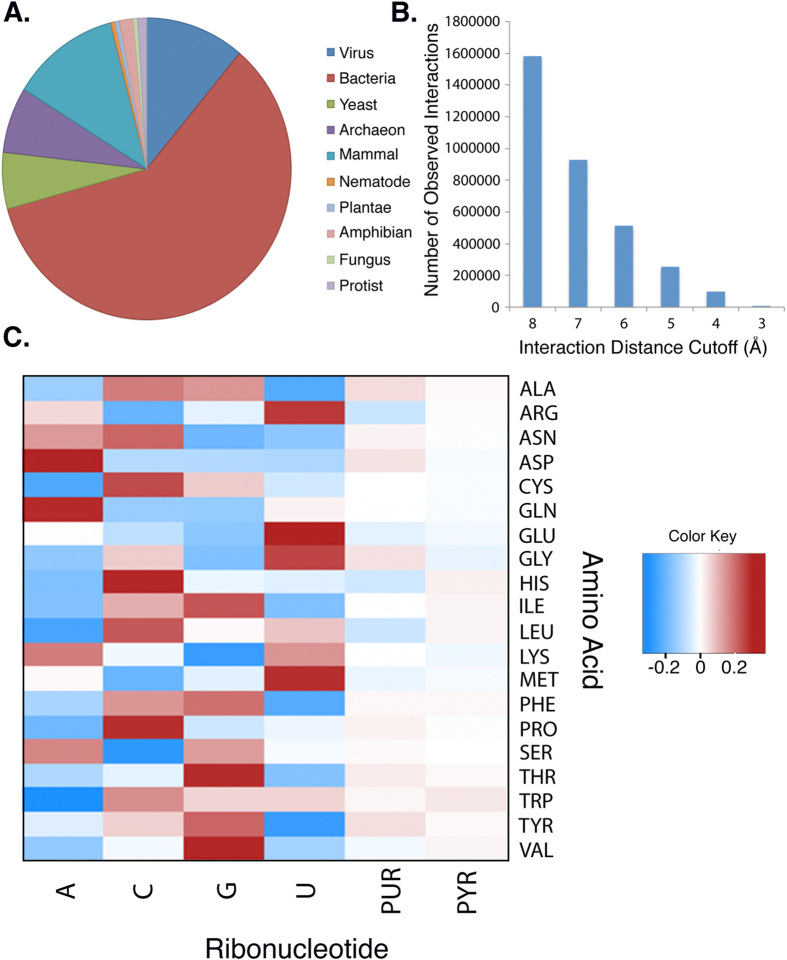
Calculating statistically inferred amino acid-nucleobase interaction preferences from known RNA-binding amino acid chains. (**A**) Composition of the 198 RNA-binding amino acid chains included in the RB199 dataset obtained from the PRIDB. (**B**) Total number of amino acid-nucleobase interactions for all RNA-Binding amino acid chains in the RB199 Dataset. (**C**) Heatmap showing calculated nucleobase interaction affinities for all possible combinations of amino acids and nucleobases. Values were calculated using a modified distance-independent contact potential formalism in which more negative values correspond to a greater affinity between an amino acid and a nucleobase.

We derived pairwise amino acid-nucleobase interaction affinities using structural information from known protein-RNA complexes. We analyzed 198 unique RNA-binding protein side chains from the RB199 dataset (Supplementary Table S1) curated by the Protein-RNA Interface Database (PRIDB)^18^,^19^. The majority of the represented RNA-binding proteins in the RB199 dataset were bacterial. The set also included; among others, viral, archaeal, fungal and mammalian ribonucleoprotein complex data (Fig. 1A). In total 1,583,881 amino acid side chain-RNA interactions were observed at a distance cutoff of 8 Å, with the number of observed interactions decreasing as the cutoff distance was decreased to 3 Å (Fig. 1B).

Amino acid-nucleobase interaction affinities were calculated using a distance-independent formalism (see methods), which compares the observed number of amino acid atoms and nucleobase atoms that are closer than 8 Å (i.e. thought to be interacting) with the probabilistically expected number of interactions. Since the number of atomic pairs in proximity was used rather than centers of geometry, amino acid-nucleobase interactions in our study are weighted by the proximity of each molecule, as closer amino acid-nucleobase pairs may have greater numbers of interatomic distances less than 8 Å. Each interaction affinity value was scaled from −1 to 1; greater affinities are associated with more negative values due to the more negative free energy of interaction between the amino acid and nucleotide. Pairwise interaction values were calculated for each amino acid-nucleobase pair (A,C,G,U-Aff) and for each amino acid-purine/pyrimidine (PUR,PYR-Aff) pair to generate an array of 120 amino acid interaction affinities (Fig. 1C; Supplementary Table S2).

### Amino Acid-Nucleobase Interactions Correspond to Respective Amino Acid Codon Content

**Figure 2:**
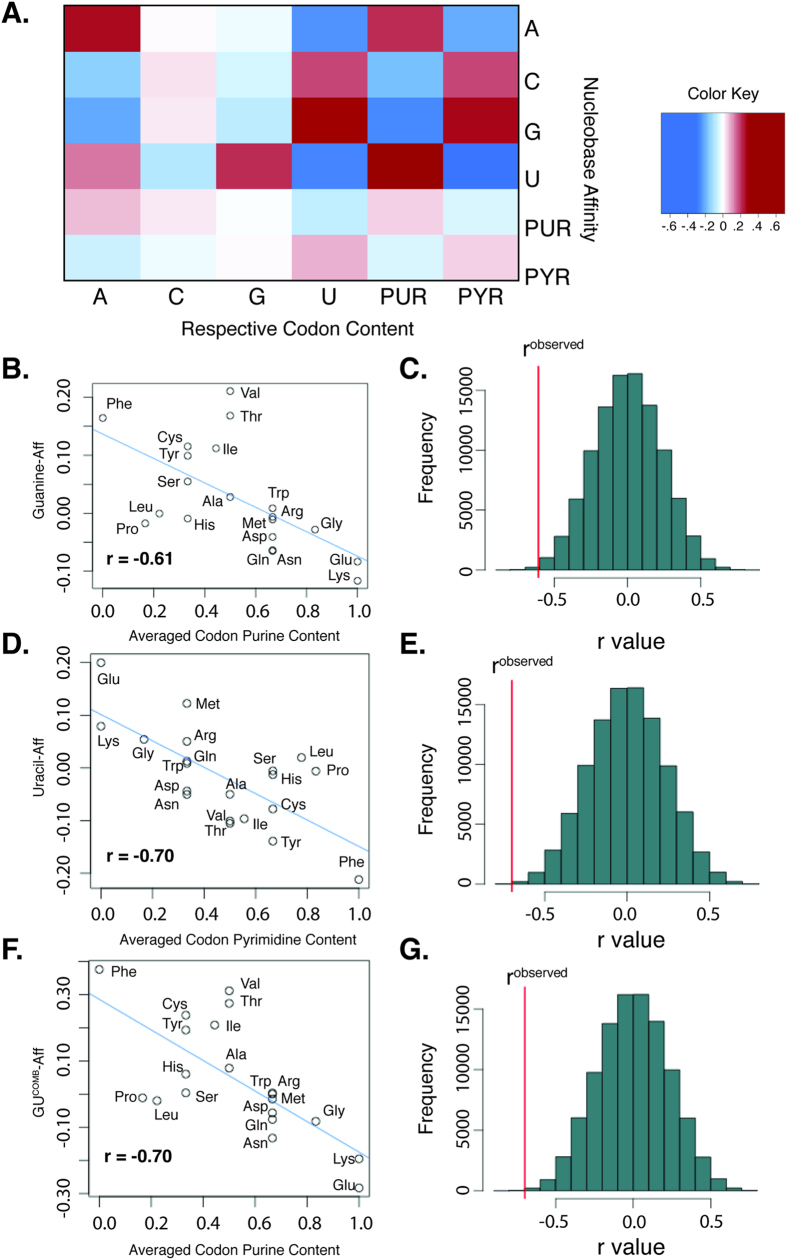
Comparing amino acid-nucleobase preferences with nucleobase content of cognate RNA codons. (**A**) Heatmap matrix showing Pearson correlation coefficients (*r*) for amino acid-nucleobase affinities and cognate amino acid codon content, negative values of correlation indicate that the relevant nucleobase-specific interaction affinity of an amino acid matches with the corresponding nucleobase content of the amino acid’s codon. (**B,D,F**) Linear regression plots showing negative correlation between amino acid G-Aff and codon purine content (*r* = −0.61, *p* < 0.005; **B**), amino acid U-Aff and codon pyrimidine content (*r* = −0.70, *p* < 0.001; **D**) and for GU^Comb^-Aff and codon purine content (*r* = −0.70, *p* < 0.001; **F**). (**C,E,G**) Histograms showing distribution of calculated *r*-values obtained following each reshuffling of amino acid-nucleobase affinities. Red line indicates the *r*-value calculated for the non-shuffled amino acid affinities for G-Aff/purine content (**C**), U-Aff/pyrimidine content (**E**) and GU^Comb^-Aff and codon purine content (**G**).

We next compared the calculated nucleobase affinities of each amino acid to the averaged mRNA composition of their cognate codons. Pearson correlation coefficients (*r*) were calculated for all possible combinations of amino acid-nucleobase affinities and cognate codon nucleobase content (Fig. 2A). Statistical significance was determined using a bootstrap technique (see methods). Negative *r*-values are associated with a greater degree of matching between codon content and amino acid affinity because negative affinity values are associated with lower structurally inferred free energies of interaction.

The array of calculated correlations showed a number of interesting relationships between an amino acid’s nucleobase interaction preference and codon nucleobase content. Notably, we only observed statistically significant matching between uracil affinity and uracil content (*r* = −0.62, *p* < 0.005), indicating that amino acids with greater affinity for uracil are more likely to be encoded by uracil-rich codons. Additionally, we found statistically significant anti-matching between adenine affinity and adenine content (*r* = +0.601, *p* < 0.005), corroborating previous findings^20^,^21^. We next tested for correlations between nucleobase-specific interaction preferences and respective codon purine/pyrimidine content. Statistically significant matching was observed for guanine affinities and purine content (*r* = −0.61, *p* < 0.005; Fig. 2B,C) and for uracil affinities and pyrimidine content (*r* = −0.70, *p* < 0.001; Fig. 2D,E).

We subsequently investigated whether linear combinations of amino acid- nucleobase affinities would correlate with cognate codon nucleobase content, based on the hypothesis that any model of direct templating would incorporate multiple nucleobase affinities. In particular, we were interested in linear combinations of affinities that incorporated both purine and pyrimidine content, since direct templating would have had to account for the presence of both purines and pyrimidines. Both additive and subtractive combined interaction affinities were generated for all pairs of nucleobase affinities and the correlation between each set of combined interaction affinities and purine content was calculated (Supplementary Tables S3–S5). As our goal was to identify a combination that accounted for both the purine and pyrimidine content of corresponding codons, we focused on the interaction affinity calculated by subtracting uracil affinities from guanine affinities for each amino acid to generate GU^Comb^-Aff. We observed statistically significant matching between GU^Comb^-Aff and the purine content of respective codons (*r* = −0.70, *p* < 0.001; Fig. 2F,G).

### Observed Matching Between Amino Acid Affinities and Codon Contents are Higher For Mitochondrial Genetic Code Variants

We next tested whether observed matching is maintained in mitochondrial variants of the genetic code^22^,^23^. We repeated the *r*-value calculation for GU^Comb^-Aff values and the purine content of respective codons for the genetic codes used by vertebrate mitochondria (*r* = −0.698, *p* < 0.001; Supplementary Fig. 1A,B), invertebrate mitochondria (*r* = −0.707, *p* < 0.001; Supplementary Fig. 1C,D), and yeast mitochondria (*r* = −0.768, *p* < 0.001; Supplementary Fig. 1E,F). Intriguingly, all mitochondrial genetic codes showed either an equivalent or greater degree of matching. Additionally, the greatest matching was observed in the yeast mitochondrial genetic code, which is also the mitochondrial genetic code variant that differs most compared to the standard genetic code: a finding that may highlight the greater importance of physiochemical interactions in potentially more ancient methods of mRNA translation.

### Decoding mRNA-encoded Amino Acid Sequences Using Amino Acid-Nucleobase Affinities

We performed linear fits on the correlation of purine content to all amino acid-nucleobase affinity normalized linear combinations (of one or two amino acids) to determine equations mapping purine content to normalized affinities. For a given linear combination of affinities, mRNA coding sequences were then translated by looking at each codon’s purine content. For each mRNA codon, amino acids were assigned probabilities based on squared distance from the fit line. An amino acid was then chosen probabilistically, and this process was repeated for each subsequent codon (see methods).

**Figure 3:**
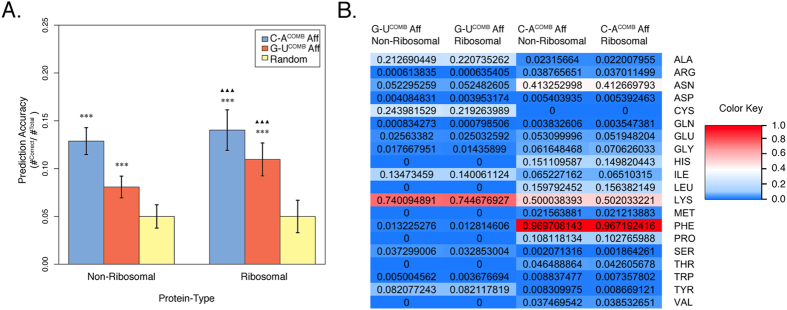
Combining amino acid-nucleobase affinities with mRNA nucleobase content to predict amino acid sequences without universal genetic code. (**A**) Bar chart showing average predictive accuracy of primary sequence predictor vs. average prediction accuracy using randomly assigned amino acids for a sample of 16,884 non-ribosomal proteins and 188 ribosomal proteins. Each amino acid sequence was predicted 100 times to determine protein-specific prediction accuracy. Two scales were used, the CA^Comb^-Aff, and the GU^Comb^-Aff. Error bars represent standard deviation. ***indicates *p* < 0.001 when compared with random, ▲▲▲ indicates p < 0.001 when compared with non-ribosomal, unpaired Student’s *t*-test. (**B**) Heatmap of the prediction accuracies, with percent accuracy separated by amino acid.

We performed this on 17,072 human mRNAs, which represent nearly all known human genes, of which 188 coded for ribosomal proteins (see methods; Supplementary Table S6). For both AC^Comb^-Aff, which had the most accurate prediction rates, and GU^Comb^-Aff, which had the highest purine content correlation, both non-ribosomal and ribosomal protein primary sequence could be predicted significantly more accurately than by chance (*p* < 0.001, unpaired *t*-test). Furthermore, ribosomal proteins could be predicted significantly more accurately than non-ribosomal proteins (*p* < 0.001, unpaired *t*-test; Fig. 3A). Prediction accuracies were also calculated separately for each amino acid (Fig. 3B). These amino acid-specific accuracies varied greatly depending on the linear combination of nucleobase affinities used, but did not differ significantly between ribosomal and non-ribosomal proteins.

Other linear combinations showed comparable results when tested on a randomly selected subset of 1,000 of the non-ribosomal proteins and all ribosomal proteins (Supplementary Table S7). These results may suggest that direct templating of ribosomal proteins occurred before other types of proteins were translated, potentially bridging the gap between the ancient “RNA world”^24^ and today’s system of mRNA translation.

### Amino Acid Interaction Affinities Mirror Cognate mRNA Sequences Across Diverse Proteomes

Our previous analyses examined correlations at the level of individual codons and amino acids. These results do not, however, preclude the previous finding^13^ that interactions between amino acids and cognate RNA could occur at the level of short stretches of the peptide chain. We therefore tested whether window-averaged amino acid-nucleobase affinities would correspond to the purine contents of cognate mRNA windows in known proteomes (see methods).

**Figure 4:**
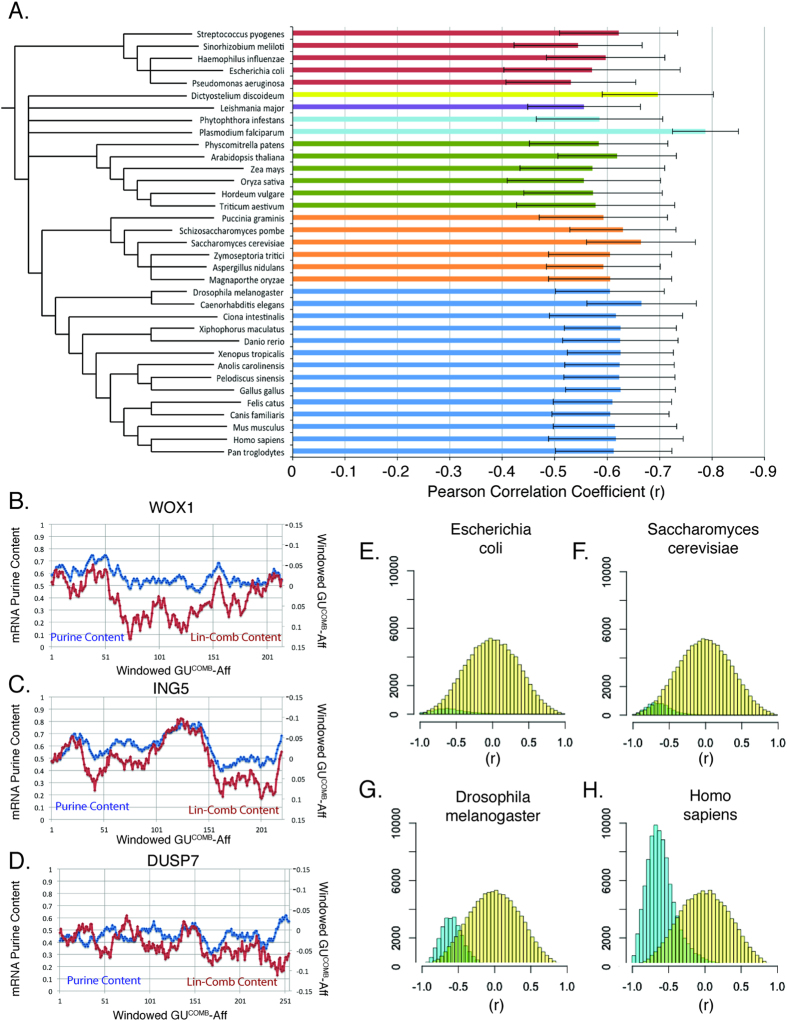
Windowed protein nucleobase affinities match corresponding mRNA nucleobase content across 35 species. (**A**) Median proteome correlation values calculated for 35 evolutionarily diverse species. Species-specific analyses all show matching but there are significant differences in the amount of anti-correlation observed (*p* < 0.001, Kruskal-Wallis one-way analysis of variance). Error bars represent median absolute deviation about the median. Phylogenetic tree created using taxonomic classifications obtained from NCBI taxonomic ID database. Bars are colored based on taxonomic clade as follows: red = Bacteria, yellow = Amoebozoa, purple = Excavata, teal = Chromalveolata, green = Plantae, orange = Fungi, blue = Animalia. (**B–D**) Sample windowed amino acid-nucleobase affinities and cognate mRNA codon content for three proteins, (**B**) WOX-1 shows average matching (*r* = 0.70), (**C**) ING5 shows greater than average matching (*r* = 0.90), (**D**) DUSP7 shows no apparent matching (*r* = 0.04). (**E–H**) Histograms showing the distributions of protein specific correlation values for windows of GU^Comb^-Aff and windows of cognate mRNA purine content for all mRNA sequences from *Escherichia coli* (**E**), *Saccharomyces cerevisiae* (**F**), *Drosophila melanogaster* (**G**) and *Homo sapiens* (**H**) versus randomized resampling distributions. The species-specific protein correlation distributions are shown in blue and null distributions generated by random CDS resampling and amino acid affinity reshuffling are shown in yellow. Differences in species-specific histogram sizes reflect differences in the relative proteome sizes of these organisms.

We calculated windowed nucleobase affinity profiles and cognate mRNA purine contents for all coding sequences (CDS) in the proteomes of 35 species (Fig. 4A). Based on our previous analysis we calculated protein specific correlations between windowed GU^Comb^-Aff and windowed purine content. Correlation values for each protein were calculated and used to derive species-specific protein correlation distributions. Three representative examples of windowed nucleobase affinity and mRNA codon content profiles for human proteins are shown: WOX-1, a putative oxidoreductase protein, showed average matching (*r* = +0.70; Fig. 4B), ING5, a p53 interacting protein implicated in tumor suppression, showed greater than average matching (*r* = +0.90; Fig. 4C) and DUSP7, a phosphatase involved in the mitogen-activated protein kinase cascade, showed no apparent matching (*r* = +0.04; Fig. 4D). The statistical significance of the observed distributions was then checked against a randomized distribution representing the null hypothesis that amino acid-nucleobase affinities do not significantly contribute to the observed correlation (see methods). Species-specific distributions are shown for *Escherichia coli* (Fig. 4E), *Saccharomyces cerevisiae* (Fig. 4F), *Drosophila melanogaster* (Fig. 4G) and *Homo sapiens* (Fig. 4H). Differences in observed distribution sizes (shown in blue) are due to variations in proteome size between the organisms examined. Our finding that matching between nucleobase affinities and cognate codon content are present in multiple species highlights the conserved nature of potential amino acid-mRNA cognate interactions (Fig. 4A). Intriguingly, however, there is a large degree of variation (*p* < 0.001, Kruskal-Wallis one-way analysis of variance) in median protein anti-correlation between species, with *Dictyostelium discoideum* and *Plasmodium falciparum* showing much greater matching than other tested species. These differences do not appear to be based on phylogenetic placement (Fig. 4A), and further work is needed to elucidate the basis of these observed differences.

## Discussion

In this study we have used structurally inferred amino acid-nucleobase interaction preferences to explore potential physiochemical interactions underlying the universal genetic code. We found that amino acid-nucleobase affinities show significant anti-correlation with corresponding amino acid codon nucleobase content. Moreover, these anti-correlations were also observed at the level of short windows of amino acids in the proteomes of evolutionarily diverse species. However, one of the drawbacks of our work and previous work is the reliance on high-resolution structural data, which is somewhat biased in favor of structured proteins and may underrepresent interactions between mRNA and intrinsically disordered regions of proteins. In the future it may be possible to extend this analysis by corroborating X-ray crystal structure data with data from mRNA interactome capture methods^25^ and even more refined techniques such as RBDmap (Matthias Hentze, personal communication).

This work follows on from and expands upon results and methodologies of previous studies examining how structurally derived nucleobase-amino acid affinities could further our understanding of the structure of the genetic code^26^. A notable difference in our methodology is that our work was performed using a database of interatomic distances as opposed to previous work conducted using molecular centers of geometry. Our findings support previous work showing significant correlation between amino acid-nucleobase affinities and cognate codon nucleobase content, and that this matching is conserved at the level of windowed stretches of mRNA and protein primary sequence^16^. However, our findings are distinct in that we observed statistically significant matching for guanine affinity and purine content and uracil content and pyrimidine content, whereas previous work identified matching between guanine affinities and purine content and cytosine affinities and pyrimidine content. The basis for this difference is not immediately clear but may be resolved by the acquisition of larger datasets of ribonucleoprotein structures.

In addition to corroborating previous work, our study also contains a number of new findings. We have shown that it is possible to use amino acid-nucleobase affinities to predict protein primary sequence from mRNA sequence significantly more accurately than by chance, which we believe supports the stereochemical hypothesis of the evolution of the genetic code^8^. Although these predictions are rough, proponents of the stereochemical hypothesis have acknowledged that any form of direct templating would be inaccurate^8^ and we would therefore expect our prediction algorithm to also be inaccurate even if the stereochemical hypothesis were true^8^. In addition, our finding that ribosomal proteins are more accurately predicted from nucleobase affinities than non-ribosomal proteins highlights the importance of ribosomal structures in deciphering the evolutionary origins of the genetic code^11^. Specifically, the capacity to better decode ribosomal machinery suggests that direct templating could play an important role in the evolution of more accurate means of mRNA translation. Our work also showed the conservation of amino acid-nucleobase affinity-codon content matching in mitochondrial genetic code variants, further emphasizing the potential evolutionary basis of observed anti-correlation.

The possibility to decode protein primary sequences using multiple combinations of amino acid-nucleobase affinities raises a number of interesting questions. Specifically, our finding that combined adenine-cysteine affinity values allow significantly more accurate decoding than combined guanine-uracil affinities is interesting given that combined guanine-uracil affinities show better matching at the level of averaged codon nucleobase content. It is possible that these findings indicate the necessity for a system of direct templating to incorporate all combinations of interaction affinities, which could be reflected by the possibility to decode primary sequences using a variety of amino acid-nucleobase affinities. Alternatively, these findings could support a hypothesis in which different amino acids are decoded using distinct combinations of nucleotide affinities.

Future work will be needed to further explore the possibility of decoding protein primary sequences using nucleobase affinities and mRNA nucleobase content. Additionally, it may also be interesting to test whether mRNA sequences can be predicted from protein primary sequences, as this would follow from Woese’s hypothesis that direct-templating could occur bi-directionally^8^. It will also be important to determine the nature of regions of primary sequence that can be predicted best and whether other classes of proteins are better predicted than average in addition to ribosomal proteins. Lastly, other types of data, such as those derived from molecular dynamic modeling experiments, will be needed to complement those based solely on X-ray crystallography and NMR experiments.

## Methods

### PRIDB Complex Analysis

This paper makes use of the RB199 dataset from the PRIDB (http://pridb.gdcb.iastate.edu/)^19^. This database consists of observed amino acid-nucleobase interactions for 198 unique RNA-binding chains (Supplementary Table S1). All structures were solved by X-ray crystallography with a minimum resolution of 3 Å. Amino acid-ribonucleobase interactions were included when the distance between atoms of a given amino acid side chain and of the RNA nucleobase was below 8 Å; this distance was previously identified as the optimal interaction distance for structurally inferring interaction affinities^27^. In total the RB199 dataset includes 1,583,881 unique observed interactions.

### Calculation of amino acid-ribonucleobase interaction preferences

An array of interaction preferences was calculated for amino acid-nucleobase pairs using a distance-independent contact potential formalism^27^,^28^,^29^


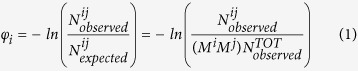


where ^*ij*^ refers to the interaction preference for a given amino acid *i* and nucleotide *j*. *N*^*ij*^_*observed*_ is the observed number of interactions below the interaction distance cutoff for *i* and *j* and *N*^*ij*^_*expected*_ is the expected number of interactions, where the expected number of interactions is the product of the molar fractions M^i^ and M^j^ multiplied by the total number of interactions N^TOT^_observed._ Our calculated amino acid interaction preferences were weighted by molecular proximities as amino acid ribonucleobase pairs that are closer together have a greater number of observed interactions. There is a negative sign in front of the logarithm to reflect the fact that pairs of amino acids-nucleotides with high affinities should be associated with negative values of estimated free energy of binding.

### Codon Content Correlation Calculation

Amino acid nucleobase affinity-amino acid codon nucleobase content Pearson correlation coefficients (*r*) were determined using R’s in-built Pearson correlation calculator. The use of the Pearson correlation coefficient was based on previous studies performing analyses of amino acid affinity-codon nucleobase content matching^16^. Amino acid codon nucleobase content was calculated by averaging the nucleobase content of all degenerate codons from either the standard genetic code or mitochondrial variants, where applicable.

The statistical significance of observed correlation values was checked using a bootstrap procedure in which nucleobase interaction affinities were randomly shuffled 100,000 times and *r* was re-calculated after each reshuffle. This reshuffling procedure produces the distribution of *r*-values given the null hypothesis that amino acid affinity values do not correlate with averaged codon nucleobase content. *p*-values were then determined by comparing the observed *r*-value to the bootstrap distribution where the *p*-value is given by:


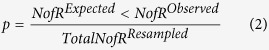


### Primary Sequence Prediction

Linear fits were calculated for the correlation of purine content to each amino acid-nucleobase-affinity linear combination. GU^Comb^-Aff was found to be:


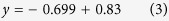


where *y* is GU^Comb^-Aff, and *x* is purine content. This linear fit was determined from the calculated GU^Comb^-Aff, normalized to values ranging from 0 to 1. Other linear fits were determined in the same manner.

To translate coding sequences of mRNA for a given affinity linear combination, the following was performed for each codon. The purine content was input into the linear fit equation (eq. 3 for GU^Comb^-Aff), and all *n* amino acids with an affinity within a determined distance cutoff of *y* were taken. The distance cutoff was calculated to be the minimum distance such that each amino acid could potentially be selected. For GU^Comb^-Aff, this was 0.16349. Other values were tested, including the minimum distance such that the number of amino acids within the cutoff distance was greater than 0 for each possible purine content, and yielded comparable results on a subset of the data (Supplementary Table S7). Then, for a given amino acid *i* of these *n* amino acids, a probability was assigned such that


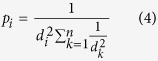


where *d*_*i*_ is the normalized affinity of the *i*^th^ amino acid minus the *y*-value output by the linear fit equation (eq. 3 for GU^Comb^-Aff). The square of the distance was used to mirror the squared distance used in the least squares regression linear fit of purine content versus the linear combination of affinities. Once probabilities were calculated using equation 4 for all *n* amino acids, one ami*n*o acid was chosen at random and weighted according to the determined probabilities. This was repeated for all codons. Finally, an accuracy value was calculated by comparing the predicted amino acids to the translated mRNA strand, and dividing the number of amino acids correctly predicted by the total number of amino acids in the translated strand. Amino acid-specific prediction accuracies were calculated in the same manner, dividing the number correct for a given amino acid in the predicted strand by the actual number of the given amino acid in the fully translated stand.

The full human proteome and corresponding mRNAs were taken from the January 2013 release of the UniProt database^30^. Only Swiss-Prot^31^ reviewed entries were used, with proteins annotated as “uncertain” excluded, resulting in the 17,083 sequences used previously^16^. Additionally, mitochondrial mRNAs were excluded from our analysis, resulting in 17,072 mRNA coding sequences. Each mRNA was translated 100 times using the outlined algorithm, and results were averaged. Of these sequences, 188 coded for ribosomal proteins; ribosomal sequences were determined by cross-referencing with the UniProtKB database (www.uniprot.org).

An unpaired, two-tailed Student’s t-test was used to determine significance of non-random as compared to random prediction accuracy and to determine the significance of ribosomal prediction accuracy compared to non-ribosomal prediction accuracy. The Student’s t-test was chosen based on the observation of approximately normal distributions of prediction accuracies.

### Proteome Correlation

Datasets containing all predicted CDS for 30 eukaryotic organisms (Supplementary table S2) were obtained from the Ensembl (www.ensembl.org) database of eukaryotic genomes^32^. Additional bacterial CDS datasets were obtained by combining predicted gene annotation files from the Microbial Genome Database for Comparative Analysis (MBGD)^33^ with the following National Center for Biotechnology Information (NCBI) provided genomic sequences: *Streptococcus pyogenes*, NC_002737.1; *Haemophilus influenza*, NC_000907.1; *Sinorhizobium meliloti*, NC_003037.1; *Escherichia coli*, NC_017906.1.

Pearson correlation coefficients were calculated for each CDS sequence by using 21 residue windows of amino acid-nucleobase preferences and the PUR or PYR content values for the corresponding window of mRNA. A window size of 21 residues was used based on previous studies of interactions between proteins and cognate mRNA sequences^16^. Statistical significance was determined by a bootstrap of interaction preference in which amino acid interaction preferences were reshuffled 100,000 times. For each reshuffle a protein was randomly sampled with replacement from the species being tested. Reported *p*-values were calculated using the non-parametric Kolmogorov–Smirnov test implementation in R. The variance of the protein correlation value distributions across species was calculated using the Kruskal–Wallis test, which assumes that tested distributions are similar in shape, which is true for our data.

### Data Visualization

Protein-RNA complex visualizations were taken from PRIDB database. Results were visualized using the statistical software R with the use of RStudio and the ggplot2^34^ visualization package.

## Additional Information

**How to cite this article**: Cannon, J. G. D. *et al.* Cross-species conservation of complementary amino acid-ribonucleobase interactions and their potential for ribosome-free encoding. *Sci. Rep.* **5**, 18054; doi: 10.1038/srep18054 (2015).

## Supplementary Material

Supplementary Information

Dataset 1

Dataset 2
