# Supplementary material for: Cross-species conservation of complementary amino acid-ribonucleobase interactions and their potential for ribosome-free encoding
Source: Sci Rep. 2015 Dec 10;5:18054. doi: 10.1038/srep18054 (PMC4674897; doi:10.1038/srep18054)
Supplement: Supplementary Information [file srep18054-s1.pdf]

Supplementary Material for:

Cross-species conservation of complementary amino acid-ribonucleobase interactions and their potential for ribosome-free encoding

Authors:

John G. D. Cannon, Rachel M. Sherman, Victoria M.Y. Wang, Grace A. Newman

Supplementary Figure 1. Comparing amino acid-nucleobase preferences with nucleobase content of cognate RNA codons in mitochondrial genetic code variants. (A,C,E) Linear regression plots showing negative correlation between (A) amino acid GU^{Comb}-Aff and codon purine content in the vertebrate mitochondrial genetic code ($r = -0.70$, $p < 0.001$), (C) amino acid GU^{Comb}-Aff and codon purine content in the invertebrate mitochondrial genetic code ($r = -0.71$, $p < 0.001$), (E) amino acid GU^{Comb}-Aff and codon purine content in the yeast mitochondrial genetic code ($r = -0.77$, $p < 0.001$). (B,D,F) Histograms showing distribution of calculated *r*-values obtained following each reshuffling of amino acid-nucleobase affinities. Red line indicates the *r*-value calculated for the non-shuffled amino acid affinities for GU^{Comb}-Aff / vertebrate mitochondrial purine content (B), GU^{Comb}-Aff / invertebrate mitochondrial purine content (D) and GU^{Comb}-Aff / yeast mitochondrial purine content (F).

Supplementary Table S1. RB199 Dataset of pairwise atomic distances for RNA-binding protein chains. To download dataset from PRIDB database see link at: http://pridb.gdcb.iastate.edu/

Supplementary Table S2. Amino acid-nucleobase affinity values calculated using equation 1. Each value refers to the interaction affinity between the amino acid on y-axis and the ribonucleotide on the x-axis.

Supplementary Table S3. Pearson correlation coefficients for nucleobase - amino acid interaction affinities, on y-axis, and respective codon contents, x-axis.

Supplementary Table S4. Pearson correlation coefficients for additive linear combinations of amino acid nucleobase affinities and respective codon purine contents.

Supplementary Table S5. Pearson correlation coefficients for subtractive linear combinations of amino acid nucleobase affinities shown on x-axis minus nucleobase affinities shown on y-axis and respective codon purine contents.

Supplementary Table S6. Protein primary sequence predictor accuracies when using varied linear combinations of amino acid-nucleobase affinities.

Supplementary Table S7. All human mRNAs used for primary sequence predictor testing.

	Adenine	Cytosine	Guanine	Uracil	Purine	Pyrimidine
**ALA**	-0.0300781	0.03543437	0.02807169	-0.0501246	0.00769161	-0.000613
**ARG**	0.00968173	-0.0370051	-0.0063126	0.05027865	-0.0129981	0.0010475
**ASN**	0.0482135	0.0720663	-0.0651131	-0.0503496	0.00685359	-0.0005464
**ASP**	0.13281233	-0.0394899	-0.0408471	-0.0439044	0.02044214	-0.001618
**CYS**	-0.3298689	0.37296367	0.11529876	-0.0780493	0.01124288	-0.0008943
**GLN**	0.14417267	-0.0617735	-0.0636869	0.01263385	0.00340917	-0.0002723
**GLU**	0.01233501	-0.0417029	-0.0836126	0.19968968	-0.0105674	0.0008505
**GLY**	-0.0236446	0.01815826	-0.0281483	0.05405059	0.01259387	-0.001001
**HIS**	-0.0419778	0.06354144	-0.0091495	-0.0127749	-0.0172817	0.00139595
**ILE**	-0.0950425	0.04872498	0.1120829	-0.0965191	-0.0092302	0.00074234
**LEU**	-0.0725712	0.06147041	-0.0004888	0.01949821	-0.0221135	0.00179093
**LYS**	0.09446464	0.00014114	-0.1164718	0.07903102	0.00869364	-0.0006924
**MET**	0.00814398	-0.0764319	-0.0107212	0.12223369	-0.0059118	0.00047461
**PHE**	-0.1135983	0.11961463	0.16445082	-0.2119855	-0.000704	5.64E-05
**PRO**	-0.0526494	0.08054806	-0.0175001	-0.0061589	0.00602333	-0.0004804
**SER**	0.06777884	-0.1147065	0.05473455	-0.005588	0.00296924	-0.0002372
**THR**	-0.0705616	-0.0273554	0.16835853	-0.1054423	0.01012425	-0.0008058
**TRP**	-0.1000611	0.03712205	0.00859529	0.00859529	-0.0051347	0.00041204
**TYR**	-0.0274953	0.02646938	0.09907863	-0.1390789	0.01662256	-0.0013183
**VAL**	-0.1179011	-0.021335	0.21080175	-0.1008302	-0.0213965	0.00173219

	A	C	G	U	PUR	PYR
**A**	0.60171696	0.00828816	-0.0588874	-0.5710329	0.48405306	-0.4840531
**C**	-0.3460103	0.0704705	-0.1214806	0.42161337	-0.4142439	0.41424392
**G**	-0.4959089	0.05223304	-0.1922637	0.66493261	-0.6095849	0.60958494
**U**	0.30675718	-0.2217055	0.48723886	-0.6228719	0.69927296	-0.699273
**PUR**	0.14608667	0.0578451	-0.0197909	-0.1813152	0.11271367	-0.1127137
**PYR**	-0.1467177	-0.0579736	0.0198779	0.1819919	-0.1131992	0.11319924

	A_Aff	C_Aff	G_Aff	U_Aff
**A_Aff**	NA	-0.1347911	0.05491633	-0.699417
**C_Aff**	-0.1347911	NA	0.63728682	-0.2226688
**G_Aff**	0.05491633	0.63728682	NA	-0.1629037
**U_Aff**	-0.699417	-0.2226688	-0.1629037	NA

	A_Aff	C_Aff	G_Aff	U_Aff
**A_Aff**	NA	-0.4756918	-0.5968827	0.13683346
**C_Aff**	0.47569185	NA	-0.1165537	0.66175767
**G_Aff**	0.59688269	0.11655375	NA	0.69804776
**U_Aff**	-0.1368335	-0.6617577	-0.6980478	NA

	Non-Ribosor	Non-Ribosor	Ribosomal A	Ribosomal Std Dev
**U-Aff**	0.0292258	0.00829209	0.02577388	0.01055593
**G-Aff**	0.05040749	0.00817881	0.04758404	0.01116194
**A-Aff**	0.06584297	0.01025115	0.06695599	0.01422281
**C-Aff**	0.15781971	0.00940063	0.13665766	0.0125165
**AU-COMB-Aff**	0.09599986	2.43E-17	0.1127137	2.92E-17
**CA-COMB-Aff**	0.16339683	0.00854119	0.19243928	0.01073154
**CU-COMB-Aff**	0.08563471	0.01012502	0.0832481	0.01529296
**GC-COMB-Aff**	0.02409491	0.00069786	0.02343314	0.00082583
**GU-COMB-Aff**	0.09615877	0.0057952	0.13400344	0.01051028
**GA-COMB-Aff**	0.10088715	0.00289746	0.09692172	0.0039306

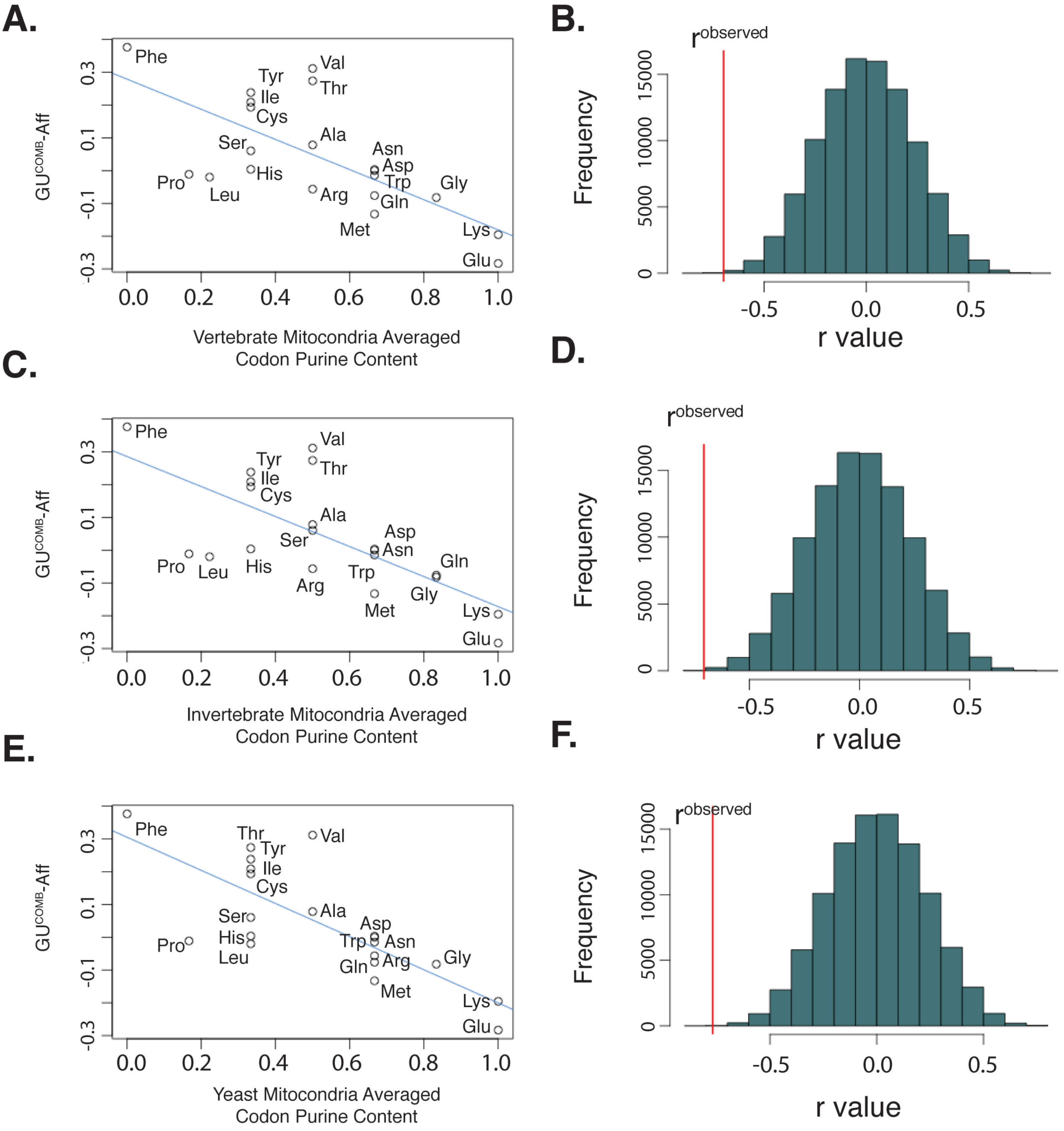
A.
B.
C.
D.
E.
F.
GU^COMB-Aff
Vertebrate Mitochondria Averaged Codon Purine Content
Invertebrate Mitochondria Averaged Codon Purine Content
Yeast Mitochondria Averaged Codon Purine Content
Phe
Val
Thr
Tyr
Ile
Cys
Ser
Ala
Asn
Asp
Trp
Gly
His
Pro
Leu
Arg
Gln
Met
Lys
Glu
0.0
0.2
0.4
0.6
0.8
1.0
-0.3
-0.1
0.1
0.3
r observed
Frequency
r value
0
5000
10000
15000
-0.5
0.0
0.5
